# DELOS Nanovesicles-Based Hydrogels: An Advanced Formulation for Topical Use

**DOI:** 10.3390/pharmaceutics14010199

**Published:** 2022-01-15

**Authors:** Lídia Ballell-Hosa, Elisabet González-Mira, Hector Santana, Judit Morla-Folch, Marc Moreno-Masip, Yaima Martínez-Prieto, Albert Revuelta, Primiano Pio Di Mauro, Jaume Veciana, Santi Sala, Lidia Ferrer-Tasies, Nora Ventosa

**Affiliations:** 1 Nanomol Technologies S.L., 08193 Cerdanyola del Vallès, Spain; lballell@nanomol-tech.com (L.B.-H.); ssala@nanomol-tech.com (S.S.); 2 Institut de Ciència de Materials de Barcelona (ICMAB-CSIC), 08193 Bellaterra, Spain; egonzalez@icmab.es (E.G.-M.); jmorla@icmab.es (J.M.-F.); marcmm97@gmail.com (M.M.-M.); albert.revu@gmail.com (A.R.); pri.dimauro@gmail.com (P.P.D.M.); vecianaj@icmab.es (J.V.); 3 Centro de Investigación Biomédica en Red-Bioingeniería, Biomateriales y Nanomedicina (CIBER-BBN), 28029 Madrid, Spain; 4 Center for Genetic Engineering and Biotechnology (CIGB), 31st Avenue between 158 and 190 Streets, Cubanacán, Playa, Havana 10600, Cuba; hector.santana@cigb.edu.cu (H.S.); yaima.martinez@cigb.edu.cu (Y.M.-P.)

**Keywords:** nanovesicles, topical drug delivery, hydrogel, rheology, vesicle integrity

## Abstract

Topical delivery has received great attention due to its localized drug delivery, its patient compliance, and its low risk for side effects. Recent developments have focused on studying new drug delivery systems as a strategy for addressing the challenges of current topical treatments. Here we describe the advances on an innovative drug delivery platform called DELOS nanovesicles for topical drug delivery. Previously, the production of DELOS nanovesicles demonstrated potentiality for the topical treatment of complex wounds, achieving well-tolerated liquid dispersions by this route. Here, research efforts have been focused on designing these nanocarriers with the best skin tolerability to be applied even to damaged skin, and on exploring the feasibility of adapting the colloidal dispersions to a more suitable dosage form for topical application. Accordingly, these drug delivery systems have been efficiently evolved to a hydrogel using Methocel^TM^ K4M, presenting proper stability and rheological properties. Further, the integrity of these nanocarriers when being gellified has been confirmed by cryo-transmission electron microscopy and by Förster resonance energy transfer analysis with fluorescent-labeled DELOS nanovesicles, which is a crucial characterization not widely reported in the literature. Additionally, in vitro experiments have shown that recombinant human Epidermal Growth Factor (rhEGF) protein integrated into gellified DELOS nanovesicles exhibits an enhanced bioactivity compared to the liquid form. Therefore, these studies suggest that such a drug delivery system is maintained unaltered when hydrogellified, becoming the DELOS nanovesicles-based hydrogels, an advanced formulation for topical use.

## 1. Introduction

Research on nanomedicine is making a revolution all over the world to offer more innovative and enhanced strategies of diagnosis and treatment compared with the conventional ones. In this frame, plenty of nanomedicines exist nowadays [1], however, the translation of a promising nanoformulation from the laboratory to the market is usually challenging and may take several years to reach its final commercialization. One of the tough challenges in this domain is to ensure the maximum quality, efficacy, and security of the drug product in the chosen administration route [2].

Among the different drug administration routes, it is of great interest to highlight that topical delivery presents several advantages since the skin is considered the most readily accessible organ, and its treatment easily allows localized drug delivery minimizing the side effects and improving the treatment efficacy. In addition, it presents patient compliance and acceptance, which is a relevant factor, compared to other administration routes. Up to now, the most common formulations for topical usages are based on drug products in semi-solid forms, which are considered almost non-invasive and easy to apply. Nevertheless, extensive research is needed to develop advanced topical drug delivery systems with improved efficacy or reduced side effects compared to the current systems. Different strategies, such as chemical enhancers, bio-polymers, and nanocarriers, among many others [3,4], are under study expecting optimized formulations for topical delivery. It should be highlighted that, in the case of the use of nanocarriers, the research of their integrity is not generally demonstrated when they are in a semi-solid form.

Hence, we present a new highly stable gellified nanovesicular system for topical delivering based on the so-called DELOS nanovesicles. These vesicles, also known as Quatsomes, are composed of a mixture of sterols and ionic surfactants, and are produced by the DELOS-SUSP platform, named Depressurization of an Expanded Liquid Organic Solution-Suspension, a compressed fluid-based method. DELOS-SUSP platform is an eco-efficient process using compressed CO_2_ that allows the reproducible and scalable production of nanovesicular systems with remarkable colloidal stability in terms of size, morphology, and lamellarity, in aqueous media [5,6,7].

Previous studies based on the development of novel nanoconjugates of DELOS nanovesicles integrating a recombinant human Epidermal Growth Factor (rhEGF) presented huge potentiality for epidermal regeneration. rhEGF was integrated into Quatsomes composed of cholesterol and the quaternary ammonium surfactant cetyltrimethylammonium bromide (CTAB), resulting in a feasible strategy for topical drug delivery such as complex wound treatment [8]. These nanoconjugates, in liquid dispersion, displayed proper physicochemical characteristics and higher efficacy in wound reepithelization when comparing it to the free protein on its own. In addition, these nanoconjugates are attractive skin nanocarrier candidates since being considered a well-tolerated liquid dispersion by this route. This has been confirmed by not only the human epidermis EpiSkin model but also by the outstanding results in terms of the cicatrization of the treated complex wounds for compassionate patient treatment [8,9]. Thus, considering the potential of DELOS vesicles in topical administration, here we propose, as a proof of concept, the possibility of transforming the DELOS nanovesicle liquid dispersion to a semi-solid dosage form as an improved pharmaceutical product for topical delivery. Indeed, the use of hydrogels for topical delivery offers several advantages, such as: (i) an enhanced drug release, (ii) excellent biocompatibility, and (iii) contributing to wound healing providing a moist environment that mimics the mechanical properties of the granulation tissue [10,11]. To this end, hydrogels were selected as a dosage form for the DELOS nanovesicles due to their versatility and good acceptance among the scientific community as a well-recognized semi-solid form used for topical delivery.

Besides their capability to act as nanocarriers for delivering small drugs and bioactive macromolecules (proteins, enzymes, RNA, etc.), DELOS nanovesicles have been also acknowledged due to the intrinsic antimicrobial properties of their membrane composition, mainly because of the presence of ionic surfactants, like quaternary ammonium surfactants, which can help to protect skin from infections [8]. Indeed, these cationic surfactants have a broad versatility of applications: in cosmetics and pharmaceutical formulations they are used as antimicrobial preservatives, and therapeutically as topical antiseptic for skin, burns, and wounds. Additionally, in industrial products they can be found as cleansers and disinfectants [12,13]. However, given their skin compatibility concerns [14], it is crucial to select those surfactants with major skin acceptability to design well-tolerable nanoformulations for topical delivery. With the same rationale, the selection of the dispersant medium for the nanovesicles is of great importance to ensure skin tolerability. A healthy skin surface has an acidic character with a pH of around 5, which plays an important role in barrier homeostasis and immune function. However, abnormalities in the integrity of the skin are attributable to a pH alteration and consequently, to an increase of infection susceptibility [15,16]. Taken together, the use of adequate dispersant media in a nanoformulation may lead to an increase in the treatment efficacy.

In the present study, we report the development of new and promising DELOS nanovesicle formulations by first exploring the preparation and characterization of the nanoformulations using different surfactants and dispersant media, essential to achieve the best skin tolerability. Then we transformed the liquid dispersion of DELOS nanovesicles into a semi-solid dosage form for being used as more efficient carriers for topical drug delivery. The resulting hydrogels were characterized by effective instrumental techniques to demonstrate the nanovesicles’ integrity in this final pharmaceutical form, which is a crucial quality aspect. Finally, to generate a first proof of concept of a hydrogel enriched with DELOS nanovesicles containing a biomolecule, an in vitro test was conducted with rhEGF loaded CTAB-DELOS nanovesicles to assess the impact of the gellification on the biomolecule activity.

## 2. Materials and Methods

### 2.1. Materials

DELOS nanovesicles: 5-Cholesten-3β-ol (Cholesterol BioChemica, purity ≥95%) was obtained from PanReac AppliChem (Barcelona, Spain). Cetyltrimethylammonium bromide (CTAB BioUltra, purity ≥99.0%) and Cetyltrimethylammonium chloride (CTAC, purity ≥ 98.0%) were purchased from Sigma-Aldrich (Madrid, Spain). Ethanol (HPLC grade) was obtained from Teknokroma (Sant Cugat del Vallès, Spain) and CO_2_ (purity ≥99.9%) was supplied by Carburos Metálicos S.A. (Barcelona, Spain). 1,1′-dioctadecyl-3,3,3′,3′-tetramethyl-indocarbocyanine perchlorate (DiI) and 1,1′-dioctadecyl-3,3,3′,3′-te-tramethyl-indodicarbocyanine perchlorate (DiD) were purchased from Life Technologies (Carlsbad, CA, USA).

The water used was pre-treated with the Milli-Q Advantage A10 water purification system (Millipore Ibérica, Madrid, Spain). Buffer histidine was prepared using l-Histidine (Sigma Aldrich, St. Louis, MO, USA). Sodium chloride (NaCl, purity ≥99.5%) was acquired from Thermo Fisher Scientific (Waltham, MA, USA), sodium hydrogen phosphate (Na_2_HPO_4_, purity ≥99.0%) and sodium phosphate monobasic dihydrate (NaH_2_PO_4_·2H_2_O, purity ≥99.0%) were acquired from Merck KgaA (Darmstadt, Germany). Buffer citrate was prepared using sodium citrate dehydrate purchased in Sigma Aldrich (St. Louis, MO, USA) and citric acid from Thermo Fisher Scientific (Waltham, MA, USA). Acetate buffer was obtained using sodium acetate (Sigma Aldrich, St. Louis, MO, USA) and acetic acid glacial purchased in Thermo Fisher Scientific (Waltham, MA, USA). Methocel^TM^ K4M was provided by Dow Chemical Co. (Midland, MI, USA).

Recombinant human epidermal growth factor (rhEGF) was produced by recombinant DNA technology using *Saccharomyces cerevisiae* and provided by the Center of Genetic Engineering and Biotechnology (La Habana, Cuba).

### 2.2. Preparation of DELOS Nanovesicles

DELOS nanovesicles were prepared by DELOS-SUSP (Depressurization of an Expanded Liquid Organic Solution-Suspension) as schematically represented in Figure S1 (Supplementary Materials) and described in refs. [8,17]. This methodology is based on first weighing the membrane components, cholesterol, and the surfactant (CTAB or CTAC) in a 1:1 molar ratio of cholesterol:surfactant and then dissolving them in ethanol. Afterward, this organic solution containing the membrane components is placed in a high-pressure vessel, at atmospheric pressure and working temperature (T_w_ = 35 °C). Liquid compressed CO_2_ is added, producing a CO_2_-expanded solution of ethanol with all the membrane components dissolved with a CO_2_ molar fraction of X_CO2_ = 0.64, a T_w_ = 35 °C and a working pressure, P_w_ = 10 MPa. Finally, the expanded organic solution is depressurized from the working pressure to atmospheric pressure over an aqueous solution that, in this work, would be the chosen dispersant media: water; 5 mM sodium citrate buffer, pH = 5; 5 mM sodium acetate buffer, pH = 5; 5 mM histidine buffer, pH = 7; and 5 mM phosphate-buffered saline (PBS), pH = 7.4. This step involves a flow of nitrogen (N_2_) at a working pressure of P_w_ = 10 MPa to plunge the CO_2_-expanded solution from the reactor, maintaining a constant pressure inside the vessel during depressurization. Then, the obtained dispersions were transferred to a container, hermetically sealed, and stored until use at 5 ± 3 °C. In all the DELOS nanovesicle preparations, the theoretical final membrane components concentration was always 5.5 mg mL^−1^ in a dispersant medium containing 10% (*v*/*v*) of ethanol.

In the case of the fluorescent-labeled DELOS nanovesicles, a carbocyanine dye such as 1,1′-dioctadecyl-3,3,3′,3′-tetramethyl-indocarbocyanine perchlorate (DiI) and 1,1′-dioctadecyl-3,3,3′,3′-tetramethyl-indodicarbocyanine perchlorate (DiD) were also dissolved with the membrane components in ethanol, achieving a total final concentration of 100 μM of dye in the final formulation. DiI/DiD-DELOS nanovesicles were prepared using CTAB as surfactant and water as media. For rhEGF-DELOS nanovesicles, the rhEGF was diluted in the aqueous solution of histidine (5 mM, pH = 7.0) to achieve a final bulk concentration of 100 μg mL^−1^ of protein after depressurization. rhEGF-DELOS nanovesicles were prepared using CTAB as surfactant.

### 2.3. Preparation of Hydrogels Enriched with DELOS Nanovesicles

To prepare the hydrogels enriched with DELOS nanovesicles, Methocel^TM^ K4M was added to DELOS nanovesicle dispersion to have a final concentration of 2% *w*/*w* of hydrogelling agent. To ensure a proper polymer dissolution, after the addition of the full amount of Methocel^TM^ K4M, the polymer was left hydrating at room temperature for a complete swelling. After that, the dispersion was gently and continuously stirred with a glass rod until complete dissolution of the hydrogelling agent, and a semi-solid form was achieved. The same procedure was used for the preparation of rhEGF-DELOS nanovesicles and DiI/DiD-DELOS nanovesicles-based hydrogels.

### 2.4. DELOS Nanovesicles and Hydrogels Characterization

#### 2.4.1. Particle Size, Polydispersity Index and Zeta Potential Assessment

The particle size distribution, polydispersity index (PDI) and zeta potential of the different nanovesicle dispersions produced were determined at 25 °C using a Zetasizer Nanoseries Instrument Nano-ZS (Malvern Instruments, Worcestershire, UK) with non-invasive backscatter optics, equipped with He-Ne laser at 633 nm. Size distribution and PDI were determined by Dynamic Light Scattering (DLS), otherwise, Electrophoretic Light Scattering (ELS) was exploited for the estimation of the apparent zeta potential. The samples (1 mL) were analyzed without any previous modification or dilution. All reported results are averages over three consecutive measurements of the same samples.

#### 2.4.2. Morphological Characterization by Cryo-Transmission Electron Microscopy (Cryo-TEM)

The morphology of the distinct types of produced DELOS nanovesicles was assessed by cryogenic transmission electron microscopy using a JEOL JEM-2011 transmission electron microscope (JEOL Ltd., Tokyo, Japan) operating at 200 kV. In the case of the DELOS nanovesicle dispersions, a small drop of the sample was directly placed on a copper grid coated with a perforated polymer film. Conversely, in the case of DELOS nanovesicles-based hydrogels, they were diluted 1:100 in water for an adequate fixation and preservation of the hydrogel sample in the grid. Afterward, the excess of the sample was removed by blotting with filter paper. Immediately after film preparation, the grid was plunged into liquid ethane held at a temperature just above its freezing point (−179.15 °C). The vitrified sample was then transferred to the microscope for analysis. To prevent sample perturbation and the formation of ice crystals, the specimens were kept cold (−196.15 °C) during both the transfer and viewing procedures. Images were recorded on a Gatan 724 CCD camera under low-dose conditions using Digital Micrograph 3.9.2. (Gatan Inc., Pleasanton, CA, USA).

#### 2.4.3. Hydrogel Microscopical Appearance

Optical microscopy was used to control the homogeneity of the hydrogels prepared. To test them, a drop of the hydrogel sample was placed between a glass slide. Then, the microscopic examination was carried out at a temperature of 20.0 ± 0.1 °C by the Olympus DP20 Microscope Digital Camera, using a five times magnification to get a proper observation of the hydrogel.

#### 2.4.4. Hydrogel Rheological Properties

The rheological characterization of hydrogels enriched with DELOS nanovesicles was determined by a HAAKE RheoStress 1 rheometer (Thermo Fisher Scientific™, Karlsruhe, Germany). Measurements were performed at 20.0 ± 0.1 °C using a configuration of a cone-and-plate geometry (plate diameter of 60 mm, and cone angle of 2° with a gap of 0.105 mm). Continuous shear investigations were performed to evaluate the shear stress (Pa) as a function of shear rate (s^−1^). This study was carried out over a shear rate of 0–50 s^−1^ for 3 min, then measurement was maintained at a constant shear rate of 50 s^−1^ for 1 min, and back to 0 s^−1^ for 3 min, to mimic similar conditions to the real topic administration of a hydrogel. Viscosity was measured by evaluating the shear stress versus the shear rate in the phase of a constant shear rate of 50 s^−1^.

#### 2.4.5. Absorption and Fluorescence Spectroscopy Measurements of Dye-Labelled DELOS Nanovesicles

Absorption spectra were recorded using a Varian Cary 5000 UV-Vis-NIR spectrophotometer (Agilent Technologies, Santa Clara, CA, USA) while for fluorescence emission and excitation spectra collection, a Varian Cary Eclipse equipment (Agilent Technologies, Santa Clara, CA, USA) was used. Fluorescence spectra were collected at 490 nm and the emission were collected at 500–800 nm for DiI/DiD-DELOS nanovesicles. In all the cases, samples were diluted 10 times in water and placed in a 1 cm quartz cuvette.

#### 2.4.6. Confocal Microscopic Characterization of Dye-Labelled DELOS Nanovesicles-Based Hydrogels

Gellified DiI/DiD-DELOS nanovesicles were imaged with a Leica TCS SP5 AOBS spectral confocal microscope (Leica Microsystems, Wetzlar, Germany) equipped with an HCX PL APO lambda blue 63.0 × 1.40 OIL UV objective (1.4 NA). To image the two dyes present in the DELOS nanovesicles, DiI and DiD, a drop of the hydrogel sample was placed on a MatTek 35 mm glass-bottom dish. The excitation wavelength was 488 and 633 nm for DiI and DiD, respectively. Detection was performed with photomultiplier tubes at specific ranges as follows: DiI was detected within the range of 540–610 nm and DiD within the 650–780 nm range. A z-stack of square images (512 × 512) at 8-bit depth were recorded at several positions for each sample using a z-step size of 0.17 µm. Finally, 3D reconstruction of the DiI/DiD-DELOS nanovesicles-based hydrogel images was generated from confocal z-stacks using the Imaris 9.2 Software (Oxford Instruments, Abingdon, UK).

#### 2.4.7. In Vitro rhEGF Biological Activity by Cell Proliferation Assay

The ability of free rhEGF and rhEGF-DELOS nanovesicles to stimulate the proliferation of BALB/c 3T3 clone A31 murine fibroblast cells (ECACC, Wiltshire, UK) was measured by a colorimetric assay [18]. Cells were seeded at a density of 1.5 × 10^5^ cells mL^−1^ in 100 μL per well of DMEM supplemented at 5% fetal bovine serum in 96-well plates. Then, cells were stressed until a quiescence state and incubated with different amounts of free rhEGF and rhEGF-DELOS nanovesicles formulated in aqueous dispersion and hydrogels after being diluted in DMEM media. In the case of hydrogels, they were previously diluted 1:10 in water for adequate pipetting. Afterward, the medium was removed, and the viable cells were determined by crystal violet staining. Finally, the plate was read at 578 nm using a Sensident Scan Microplate spectrophotometer (Merck, Darmstadt, Germany). The results were expressed as international units (IU), compared to a working reference material that was calibrated against the 91/530 EGF standard (NIBSC, Hertfordshire, UK). The biological activity of free rhEGF and rhEGF-DELOS nanovesicles was assessed from the absorbance at 578 nm using parallel line assay statistical software based on the stated methodology [19]. The specific biological activity of rhEGF in cell proliferation assay is defined by Equation (1).

(1) $$\mathrm{Specific}\;\mathrm{rhEGF}\;\mathrm{bioactivity}\;\left(\mathrm{IU}\cdot {\mathrm{mg}}^{-1}\right)=\left(\frac{\mathrm{cell}\;\mathrm{proliferation}\;\mathrm{assay}\;\left(\mathrm{IU}\cdot {\mathrm{mL}}^{-1}\;\right)}{\mathrm{rhEGF}\;\mathrm{nominal}\;\mathrm{concentration}\;\left(\mathrm{mg}\cdot {\mathrm{mL}}^{-1}\right)}\right)$$

### 2.5. Statistical Analysis

Statistical significance among groups was analyzed by one-way analysis of variance (ANOVA), followed by Tukey’s multiple comparison post-test. A value with *p* < 0.05 was considered statistically significant, whereby symbols indicating statistical significance were: * *p* < 0.05, ** *p* < 0.01 and *** *p* < 0.001. The statistical analyses were performed using Minitab-17 Statistical Software Package (Minitab Inc., State College, PA, USA).

## 3. Results and Discussion

### 3.1. Development of DELOS Nanovesicle Suspensions with Different Surfactant Counterions and Dispersant Media—Preparation and Physicochemical Characterization

**Figure 1:**
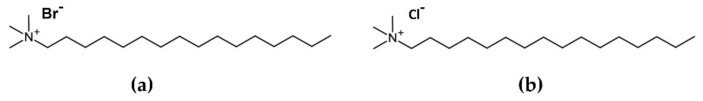
Chemical structure of (**a**) CTAB and (**b**) CTAC surfactants.

To design a wider range of potential DELOS nanovesicle formulations suitable for topical delivery, two key parameters were evaluated. First, we examined the impact of different compositions on the nanovesicles by changing the membrane components of the nanoformulation such as the quaternary ammonium surfactant. In the formulation based on DELOS nanovesicles integrating rhEGF, cetyltrimethylammonium bromide (CTAB) was the selected surfactant, which is composed of a 16 carbons hydrophobic chain with a charged ammonium polar head and bromide as a counter ion. It is important to note that CTAB is a surfactant broadly used in several cosmetic products and has demonstrated antibacterial properties that can play an important role in skin formulations avoiding wound infections [20,21,22]. Though the approach here proposed is based on the use of a similar surfactant using chloride as counterion, the major ion of the human body, named cetyltrimethylammonium chloride (CTAC), which has also been demonstrated to possess an antimicrobial effect (Figure 1 shows the chemical analogy between CTAC and CTAB). The medical regulatory agency FDA (U.S. Food and Drug Administration) has approved CTAC for topicals [23], and has reported this ingredient as being commonly found in lotions at 0.2% *w*/*w*, so indicating its safeness at this concentration [24,25,26]. Besides, to note that in previous work it was demonstrated that cationic surfactants nanostructured in the nanovesicles broadly reduced their potential irritation effect [8].

Therefore, assessing the effect of the chloride as the counterion in DELOS nanovesicle production would allow the generation of a new DELOS nanovesicle formulation as another possible optimum alternative for the dermal drug delivery.

On the other hand, it is important to emphasize that in addition to the composition of the vesicle itself, dispersant media is crucial to provide stability to the formulation, which must safeguard biocompatibility and be suitable for topical administration. Thus, besides the surfactant nature, different dispersant media in the DELOS nanovesicles systems were evaluated according to the need of pursuing more skin-tolerability of the nanoformulations. The five dispersant media chosen for being studied were: water; sodium citrate buffer (citrate buffer), pH = 5.0; sodium acetate buffer (acetate buffer), pH = 5.0; histidine buffer, pH = 7.0; and phosphate-buffered saline (PBS), pH = 7.4. All buffers were prepared at 5 mM concentration to compare all of them to the buffer used in the previously reported nanoformulation of DELOS nanovesicles integrating a recombinant human Epidermal Growth Factor, which was 5 mM histidine buffer (pH = 7.0), for complex wound healing [8]. The histidine buffer has also been reported as the most common buffer of commercially available protein therapeutics, like antibody formulations, with histidine concentration ranges from 3 mM to 50 mM and pH = 5.5–6.5 [27,28,29].

Water was used as a reference medium and as a model to prepare DELOS nanovesicles formulations [6,30]. On the other hand, histidine is an essential and neutral amino acid that presents anti-inflammatory and antioxidant properties. It has been used as a therapy to treat some skin diseases like atopic dermatitis and it can be easily combined with proteins or other amino acids and treated as a supplement for a large number of disorders [31,32]. Furthermore, recent results suggest that L-histidine has the potential to facilitate wound healing in both in vitro and in vivo models in aging skin [33]. At the same time, this medium has been used in rhEGF-DELOS nanovesicles conjugates for complex wound treatment [8,34]. Consequently, this buffer is convenient when a pH near 7.0 is requested for the treatment of a skin disorder. Conversely, sodium citrate buffer at pH = 5.0, which is the healthy skin pH [35,36,37], contains citric acid, which is a metabolic substance found in animals and plants. It is highly used in cosmetics not only as a chelating agent but also as a pH adjuster, and as a fragrance ingredient. Sodium citrate buffer is reported to be no dermal irritant up to 5% *w/v* in aqueous, and it is suitable for skin diseases and wound healing treatment [37,38,39,40,41]. Regarding acetate buffer at pH = 5.0, it contains sodium acetate, which is an interesting molecule associated with esterase metabolites present in the skin and so, it is commonly used in cosmetics as a fragrance ingredient and buffering agent due to its skin tolerability [42]. Finally, PBS is a commonly used buffer in biological research based on a salt solution containing sodium chloride, sodium hydrogen phosphate, and sodium phosphate monobasic that balances the salt concentration around cells, preventing osmosis and, thus, it is suitable for skin usages [43].

To evaluate all of these parameters, DELOS nanovesicles were prepared using the eco-efficient DELOS-SUSP methodology (see Materials and Methods section), since it is a CO_2_-based method that permits in a single step procedure a very high control of the molecular assembly to obtain nanovesicles with high homogeneity in terms of size and morphology, good batch-to-batch reproducibility, and is also scalable and compatible with GMP regulation. With this in mind, in this investigation, we seek to evaluate a broad array of possible nanoformulations for use in topical drug delivery by studying the use of different quaternary ammonium surfactants changing the counterion (CTAB and CTAC) and all the dispersant media at different pH described above (water, sodium citrate, sodium acetate, histidine, and PBS) as summarized in Table 1.

As shown in Table 1, 10 different nanoformulations have been prepared in this work performing replicates of each of them. In all the cases, the final membrane components concentration was 5.5 mg mL^−1^ with a theoretical equimolar ratio between cholesterol and surfactant. It should be pointed out that all the DELOS nanoformulations contain 10% *v*/*v* of ethanol coming from the DELOS-SUSP procedure. The presence of this amount of EtOH may be beneficial since its positive value is known as an enhancer for skin treatments [44].

**Figure 2:**
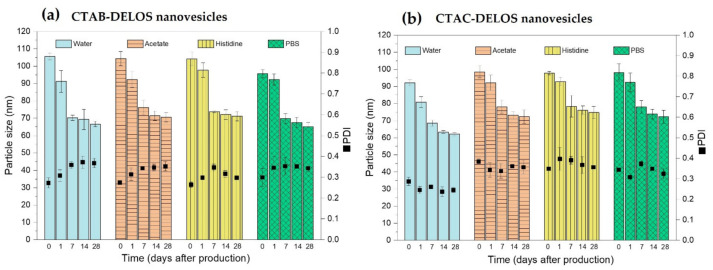
(**a**) Evolution of CTAB-DELOS nanovesicles and (**b**) CTAC-DELOS nanovesicles over time on particle size and polydispersity index (PDI) with the effect of different dispersant media: water (blue); 5 mM acetate, pH = 5.0 (orange, horizontal line); 5 mM histidine, pH = 7.0 (yellow, vertical line); and 5 mM PBS, pH = 7.4 (green, square).

Particle size and Polydispersity Index (PDI) of the nanoformulations were evaluated using Dynamic Light Scattering (DLS) over time. From Figure 2, it can be observed that planned comparisons of the use of CTAB and CTAC surfactant in the DELOS nanovesicle formulations revealed that both surfactants lead to similar mean nanovesicle size in the different evaluated dispersant media. Additionally, proper colloidal stability of the nanovesicles was observed regarding the screening of dispersant media based on water, acetate buffer (pH = 5.0), histidine buffer (pH = 7.0), and PBS buffer (pH = 7.4). As observed in Figure 2, there was a tendency to a mean particle size reduction in all the nanoformulations during the first week and stabilization at least over a month, which was the evaluated period (Figure S2, Supplementary Materials). This size tendency and the great stability of this type of vesicular system have been widely described previously in our group. It could be hypothesized that this tendency of particle size reduction over time is correlated to the thermodynamical aspects of the vesicles. DELOS nanovesicles have shown to be dynamic systems that evolve to an equilibrium state over time by minimizing the free energy of the system by the formation of small and energetically stabilized vesicles [45]. For instance, extremely long-term stability of at least 1000 days at 4 °C for CTAB-DELOS nanovesicles in water with 10% *v*/*v* of EtOH was already proved [9].

Conversely, regarding the use of citrate buffer (pH = 5.0), it was the only notable exception in this study since it showed immediate sedimentation of vesicle membrane components in all formulations after one day of production (Figure S3, Supplementary Materials). This phenomenon was also detected by the zeta potential measurement, which indicates the degree of repulsion between the charged particles in the dispersion. High positive and negative zeta potential values, i.e., >+30 mV or <−30 mV, indicate highly charged particles, which avoids particle aggregation due to electric repulsion. However, with a small zeta potential value between +30 mV and −30 mV, attraction overcomes repulsion, and it is expected that the sample first aggregates and afterward can evolve to sedimentation [46]. Zeta potential measurements of all the obtained nanoformulations are displayed in Figure S3, (Supplementary Materials). As shown, all the nanoformulations prepared with water, acetate, histidine, and PBS buffers present a zeta potential value above +100 mV, although quite a lower value of zeta potential was observed in acetate and PBS as compared with histidine and water for both CTAB- and CTAC-DELOS nanovesicles (Figure S4, Supplementary Materials). However, when these vesicles are prepared in citrate buffer, they present a zeta potential value below +30 mV. This interesting finding could suggest that a strong electrostatic interaction between the positive charge of the quaternary ammonium surfactant and the negative charge coming from the citrate molecule occurred hindering the vesicle formation, provoking aggregation, and sedimentation of the system. Therefore, in this work the formation of CTAB- and CTAC-DELOS nanovesicles was discarded using citrate buffer (5 mM pH = 5.0) as media.

**Figure 3:**
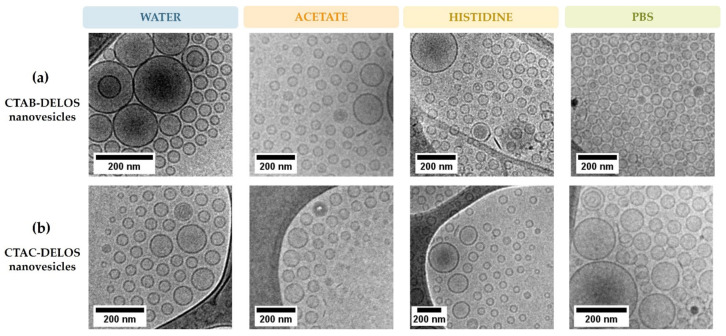
Cryo-TEM images of (**a**) CTAB-DELOS nanovesicles and (**b**) CTAC-DELOS nanovesicles in different dispersant media: water (blue); 5 mM acetate, pH = 5.0 (orange); 5 mM histidine, pH = 7.0 (yellow); and 5 mM PBS, pH = 7.4 (green).

Regarding the size and morphology of the obtained nanovesicles, they were also studied using cryogenic transmission electron microscopy (cryo-TEM). Similarly shaped nanovesicles were observed, as seen in Figure 3 in the prepared CTAB- and CTAC-DELOS nanovesicles formulations in water, and in acetate, histidine, and PBS buffers. It is remarkable to appreciate that there are almost no variation differences in the morphology of the vesicles when changing neither the surfactant, CTAB, or CTAC, nor the dispersant medium (Table 1). Indeed, most of the vesicles are homogeneous unilamellar nanovesicles with spherical morphology, with a size range of ≈50–100 nm in diameter, similar to the one reported in the literature [8,47,48,49].

Thanks to these favorable physicochemical properties of DELOS nanovesicles, we can suggest that either the use of CTAB or CTAC surfactant, with bromide and chloride counterion respectively, and the presence of water; or acetate; histidine; or PBS buffers in the nanoformulation are promising components to formulate DELOS nanovesicle liquid dispersions which are more tolerable for skin application. It should be highlighted that these nanoformulations have the potential to be translated into other types of treatments requiring these formulation characteristics regarding pH or composition.

### 3.2. Semi-Solid Dosage Form: Hydrogels Enriched with DELOS Nanovesicles

#### 3.2.1. Preparation and Physicochemical Characterization of DELOS Nanovesicles-Based Hydrogels

To develop DELOS nanovesicles-based hydrogels, the most promising aforementioned formulations, CTAB and CTAC-DELOS nanovesicles dispersed in water, acetate, histidine and PBS, were gellified by adding hydroxypropyl methylcellulose, also called Methocel^TM^ K4M. This biopolymer is a cellulose-based and water-soluble gelling agent used in injectable formulations and transdermal films or gels, giving a broader range of applicability to DELOS nanovesicles [50,51]. This polymer was also selected due to its non-ionic charge, which is an important parameter for evading electrostatic interactions with the vesicles and, thus, the possible aggregation formation.

The hydrogels were prepared using 2% *w*/*w* of the hydrogelling agent (see details in Materials and Methods section). This concentration of hydrogelling agent was selected after testing a broad range of hydrogelling agent concentrations (1–4% *w*/*w*), which are concentrations commonly used to prepare topical formulations [52]. In these preliminary studies (data not shown), this 2% *w*/*w* concentration resulted to be the optimum one to yield DELOS nanovesicles-based hydrogels with the desired consistency for dermal application and, importantly, to be easily spread in ulcers or damaged skin. Since the Methocel is a viscosity-increasing agent, lower concentrations (<2%) of hydrogelling agent yield systems with a rheological behavior similar to the aqueous dispersion, which would favor the formulation drainage from the applied region. On the other hand, higher concentrations (>2%) of the hydrogelling can favor the formation of aggregates and, thus, can jeopardize the stability of the formulation.

Once hydrogels were prepared, they were examined macroscopically. Colorless and homogeneous semi-solid formulations, with the appropriate consistency, were yielded showing, thus, excellent organoleptic properties.

**Figure 4:**
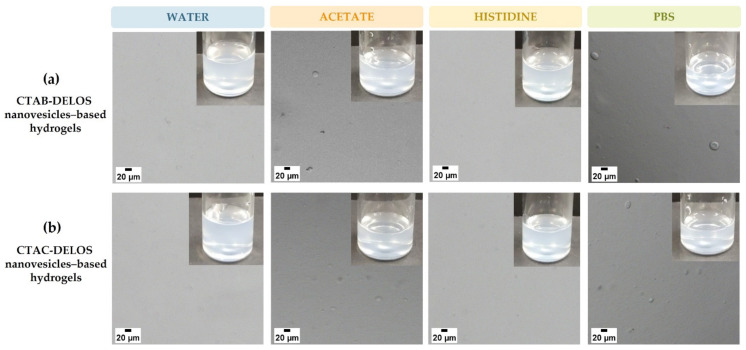
Optical microscopy and macroscopic images of Methocel^TM^ K4M hydrogels of (**a**) CTAB-DELOS nanovesicles and (**b**) CTAC-DELOS nanovesicles in different dispersant media: water (blue); 5 mM acetate, pH = 5.0 (orange); 5 mM histidine, pH = 7.0 (yellow); and 5 mM PBS, pH = 7.4 (green).

To evaluate in more detail the formulated hydrogels, they were studied by optical microscopy. Figure 4 illustrates the microscopic appearance of the hydrogels using the different quaternary ammonium surfactants and dispersant media. Hydrogels enriched with DELOS nanovesicles dispersed in water or histidine medium did not show any microscopic appearance alteration. However, particles in the micrometric range were observed in those semi-solid formulations enriched with DELOS nanovesicles that contained sodium acetate and PBS, probably related to the negative charge of acetate and phosphate anions of these buffers and its electrostatic interactions with the cationic surface charge of the DELOS nanovesicles, as suggested by the small decrease of the zeta potential of both CTAB- and CTAC-DELOS nanovesicles in acetate and PBS dispersant media (Figure S3, Supplementary Materials).

**Figure 5:**
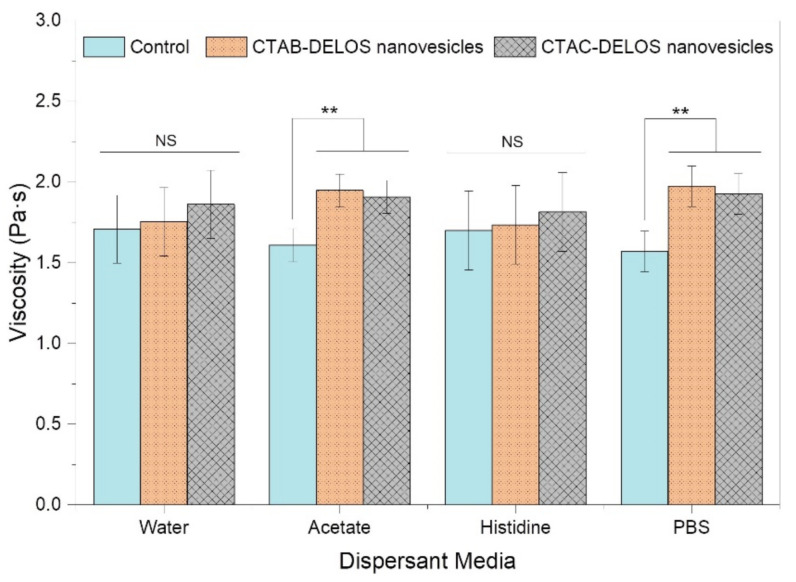
Viscosity measurements of the evaluated Methocel^TM^ K4M hydrogels, controls (samples without the nanovesicles) and DELOS nanovesicles-based hydrogels using CTAB- (orange, points) or CTAC-DELOS (grey, squares) nanovesicles in water, acetate, histidine, and PBS (*N* = 3). Data plotted as mean viscosity are represented by the heights of bars with 95% confidence intervals represented by the error bars. Statistical analyses were performed by one-way ANOVA followed by Tukey pairwise comparison. ** *p* < 0.01 and NS: non-significant differences.

The viscosity profile of the hydrogels is linked to the polymeric network and strength of constituent interactions. The viscosity and the rheological measurements performed with the formulated DELOS nanovesicles-based hydrogels provided key information on the three-dimensional network properties as influenced by the presence of different surfactant counterions and dispersant media. The viscosity of the control hydrogels (when non enriched with DELOS nanovesicles) did not show statistically significant differences among the different dispersant media evaluated (ANOVA *p*-value = 0.300) (Figure 5).

Viscosity measurements contributed to comprehending the behavior of the hydrogels when containing DELOS nanovesicles. As it can be seen in Figure 5, changes in the dispersant medium of the CTAB- and CTAC-DELOS nanovesicles resulted in different viscosity values of the obtained hydrogels. The use of water and histidine buffer with pH = 7.0 in the hydrogels showed similar results with the presence and absence of DELOS nanovesicles with non-statistically significant differences. However, it was also apparent that the addition of salts like acetate buffer with pH = 5.0, and PBS buffer with pH = 7.4 provided a statistically significant viscosity increase compared to the control. This increase could be related to the capacity of these salts (probably the presence of acetate and phosphate anions) to induce the presence of micrometric particles in the original formulation of DELOS nanovesicles, as already observed microscopically. It is reported that semi-solid dispersions with a constant solid volume fraction may differ in their viscosities due to particle size and size distribution (PDI) differences [53]. Viscosity changes can also provide information about intermolecular interactions [54]. With regard to the use of the two different quaternary ammonium surfactants, CTAB or CTAC, in the nanovesicles formulation when being gellified, no differences in viscosity were found.

Focusing on the viscosity value of the hydrogels, which is a critical quality attribute, we can observe that all of them present a value coherent with the appropriate consistency for topical application, and in line with viscosities reported in the literature for topical formulation products [55].

**Figure 6:**
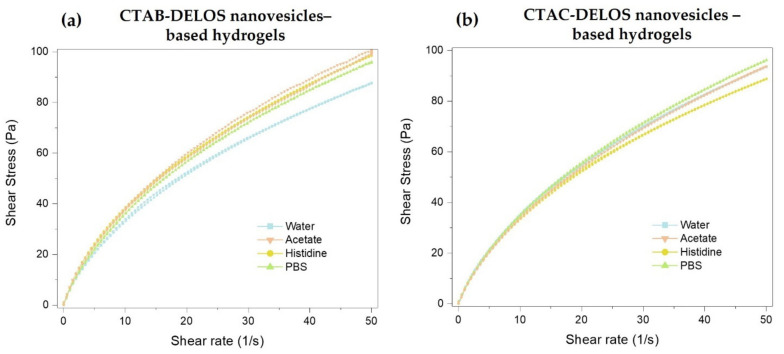
Shear rate as a function of shear stress of hydrogels enriched with (**a**) CTAB-DELOS nanovesicles and (**b**) CTAC-DELOS nanovesicles in different dispersant media: water (blue ■); 5 mM acetate, pH = 5.0 (orange ▼); 5 mM histidine, pH = 7.0 (yellow ●); and 5 mM PBS, pH = 7.4 (green ▲).

On the other hand, rheological characterization of the developed DELOS nanovesicles-based hydrogel formulations is useful to assess the flow behavior of the semi-solid systems. The flow curves of DELOS nanovesicles-based Methocel^TM^ K4M hydrogels revealed non-Newtonian pseudoplastic flow behavior. Figure 6 displays the shear stress plotted versus shear rate for all the prepared DELOS nanovesicles-based Methocel^TM^ K4M hydrogels. In all the cases, the viscosity of the gel, which is the slope of the curve, decreased with shear increasing. This pseudoplastic behavior result is a desired property for topical products since it indicates that the product can be easily spread under mild forces and so, enabling a film formation on the skin surface. This shear-induced thinning is a very appropriate characteristic for products intended to be applied to ulcers or damaged skin. However, once the shear stress is removed, it is desired that the viscosity of the formulation increases rapidly to avoid leakage [56]. This desired low or absence of thixotropy has been observed in all the samples, which presented a small or null hysteresis loop area. No impact was found on rheological behavior when hydrogels contained CTAB or CTAC, or the different dispersant media.

Although further studies are needed to understand deeply DELOS nanovesicles-based Methocel^TM^ K4M hydrogels, results obtained from previous experiments such as the microscopical analysis permitted to select the nanoformulations containing water and histidine buffer as the optimal ones to be formulated as hydrogels for topical drug delivery.

#### 3.2.2. Integrity of DELOS Nanovesicles When Gellified

**Figure 7:**
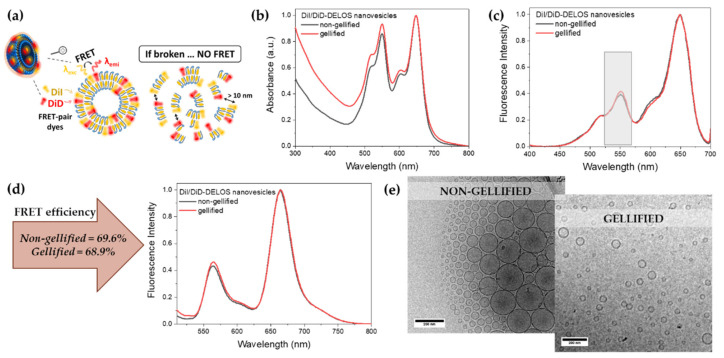
*(***a**) Representative image of the presence or absence of FRET phenomena in the whole vs. dissociate DiI/DiD-DELOS nanovesicles, respectively; (**b**) absorption and (**c**) excitation spectra of gellified and non-gellified DiI/DiD-DELOS nanovesicles; (**d**) emission spectra of gellified and non-gellified DiI/DiD-DELOS nanovesicles with their FRET efficiency value; and (**e**) cryo-TEM images of DiI/DiD-DELOS nanovesicles non-gellified and gellified. DiI/DiD-DELOS nanovesicles were prepared using CTAB as surfactant and water as media. Gellified nanovesicles were previously diluted 1:100 in water for an adequate fixation and preservation of the hydrogel sample in the grid.

It is worth noting that by the process of gellification, the characteristics of the nanovesicle medium are changed (i.e., viscosity), thus the integrity of the vesicles during the process and afterward might be compromised. To study if the characteristics of the nanovesicles are preserved once they are in the gel, mainly its integrity as a nanostructure, we exploited fluorescence spectroscopical techniques. The integration of dyes in similar DELOS nanovesicles dispersed in water as one of the selected media has been widely studied by N. Ventosa and collaborators [57]. Previously, vesicles with 1,1′-dioctadecyl-3,3,3′,3′-tetramethyl-indocarbocyanine perchlorate (DiI) and 1,1′-dioctadecyl-3,3,3′,3′-tetramethyl-indodicarbocyanine perchlorate (DiD) fluorophores were successfully prepared [58]. Importantly, DiI and DiD is a well-known Fluorescence Resonance Energy Transfer (FRET) pair [59,60,61], thus, when those dyes are nearby (typically <10 nm) they experiment high FRET efficiencies, providing information about the distance between both fluorophores. This spectroscopic tool, also known as spectroscopic ruler, is very useful to determine nanovesicle stability and integrity [62] since FRET emission fluorescence would vanish when nanovesicles dissociate or break down (see Figure 7a).

Importantly, DiI and DiD are amphiphilic molecules whose incorporation into DELOS nanovesicle membrane is driven by hydrophobic interactions and its leakage once entrapped at the membrane is negligible [57,63]. On that basis, CTAB-DELOS nanovesicles loaded with DiI and DiD were prepared, showing no differences in the size and morphology of the vesicles (Figure S5a,b, Supplementary Materials). Afterwards, they were gellified to evaluate the stability and integrity of the nanovesicles through the fluorescence emission of the dye pair (Figure S5c, Supplementary Materials).

First, absorption spectra of nanovesicles loaded simultaneously with DiI and DiD were recorded in both conditions, in liquid dispersion and in gel (Figure 7b). Absorption spectra revealed that nanovesicles either in liquid dispersion or in gel maintain DiI and DiD molecules in the nanovesicles nanostructure since no change in the main absorption bands (Abs(DiI) = 551 nm and Abs(DiD) = 650 nm) are observed. It is worth noting that DiD is prone to form aggregates such as H-dimers, generally reported by an increase in the absorption shoulder at 600 nm [58,64]. However, spectra displayed in Figure 7b point out the lack of those aggregates or any other non-monomeric forms of DiD. Thus, with that information, we can first conclude that nanovesicles seem to maintain their membrane stability when transferred to the gel. Secondly, FRET efficiency between DiI and DiD, as a donor and acceptor respectively, was interrogated through excitation and emission fluorescence spectroscopy. Planned comparisons of the gellified and non-gellified groups revealed that the excitation spectra of the dyes are similar among the groups (Figure 7c). More importantly, excitation spectra also indicated the presence of FRET phenomena since DiI (donor) is excited when probing the DiD (acceptor) emission at λem = 710 nm (Figure 7c, highlighted excitation band in grey). Indeed, the FRET efficiency was estimated through the ratiometric approach from emission spectra upon excitation at 490 nm using the following Equation (2) [65]:

(2) $$\mathrm{Efficiency}\;\mathrm{FRET}\;\left(E_{\mathrm{FRET}},\;\%\right)=\frac{\mathrm{Intensity}\;\mathrm{DiD}\;}{\mathrm{Intensity}\;\mathrm{DiI}+\mathrm{Intensity}\;\mathrm{DiD}}$$

Two emission bands appeared at ∼569 and ∼673 nm corresponding to the DiI and DiD emission, indicating again that both dyes are stably entrapped at the nanovesicle membrane indistinctly of its medium. The estimation of FRET efficiency in the non-gellified and gellified DiI/DiD-DELOS nanovesicles resulted in similar values of practically 70% (Figure 7d). Finally, the morphology of DiI/DiD-DELOS nanovesicles-based hydrogels was observed by the cryo-TEM technique. As observed in Figure 7e, this analysis indicates that the morphology of these DELOS nanovesicles was not altered when being gellified with Methocel^TM^ K4M at 2% *w*/*w*, suggesting again their stability in the hydrogel.

Taking all these results together, it is clear that the gellificant does not impact the DELOS nanovesicle structure, from the spectroscopic data but also from the cryo-TEM observation. Spectroscopic measurements from DiI and DiD in nanovesicles-based hydrogels demonstrating the preservation of the FRET emission are indicative of the membrane integrity.

Finally, DiI/DiD-DELOS nanovesicles-based hydrogels were examined by confocal fluorescence microscopy to evaluate the distribution of the two dyes in the hydrogel. During the analysis, DiI and DiD emission signals were recorded showing colocalization of the two dyes, meaning an overlap of them in the final image due to their close position in the structure. As we can see in Figure S6 (Supplementary Materials), there is a significant presence of yellow points representing the colocalization of the two dyes used, DiI (in green) and DiD (in red), on the same region. This phenomenon emphasizes that the two dyes associate with the same vesicle structure and so, suggesting again the integrity of the vesicles when gellified with 2% *w*/*w* Methocel^TM^ K4M. Additionally, the magnification of the confocal image reveals the abundance of dispersed particles with a particle size smaller than 1 µm, suggesting that DELOS nanovesicles are mostly not aggregated in the hydrogel.

Overall, it is worth mentioning that all of these experimental results go beyond extensive literature, demonstrating the integrity of the DELOS nanovesicle formulations when being gellified, which is an important finding in understanding their behavior.

#### 3.2.3. In Vitro Protein-Specific Bioactivity of Gellified rhEGF-DELOS Nanovesicles

**Figure 8:**
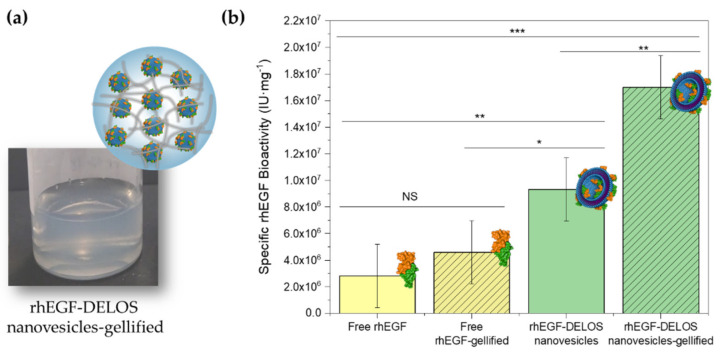
*(***a**) Macroscopic image of the rhEGF-DELOS nanovesicles-based hydrogel using CTAB as surfactant and 5 mM histidine, pH = 7, as media; (**b**) in vitro specific biological activity of free rhEGF and rhEGF-DELOS nanovesicles in a cell proliferation assay in 3T3 A431 murine fibroblast cell line. Free rhEGF and rhEGF-DELOS nanovesicles 100 μg mL^−1^ were formulated in aqueous solution or in Methocel^TM^ K4M hydrogel. Data plotted as mean (*n* = 3 per group) specific bioactivities are represented by the heights of bars with 95% confidence intervals represented by the error bars. Statistical analyses were done by one-way ANOVA followed by Tukey pairwise comparison. * *p* < 0.05, ** *p* < 0.01, *** *p* < 0.001 and NS: non-significant differences.

The production of nanoconjugates based on the integration of recombinant human Epidermal Growth Factor (rhEGF) in DELOS nanovesicles demonstrated potentiality for the topical treatment of complex wounds, achieving well-tolerated liquid dispersions by this route [8,9]. As a further step towards achieving a semi-solid dosage form as a new final pharmaceutical product with better performances, CTAB-DELOS nanovesicles integrating rhEGF in histidine buffer (5 mM, pH = 7) were successfully gellified with 2% *w*/*w* of Methocel^TM^ K4M, presenting coherent properties in terms of macroscopic appearance, viscosity values, and rheology behavior compared to the previously described hydrogels using the same nanoformulation but without rhEGF (Figure 8a). Besides, as observed in Figure S7 (Supplementary Materials), the morphology of rhEGF-DELOS nanovesicles was not altered when being gellified, suggesting again their stability in the hydrogel.

In order to study the impact of the gellification in the rhEGF specific bioactivity, in vitro measurements were performed comparing free and integrated rhEGF in CTAB-DELOS nanovesicles both in liquid dispersion and gellified. Prior to the experiment, it was assessed whether the Methocel^TM^ K4M hydrogelling agent could interfere in the rhEGF colorimetric assay for the determination of the biological activity of the rhEGF. To do it, a placebo hydrogel (without both, nanovesicles and rhEGF) and a hydrogel with CTAB-DELOS nanovesicles (nanovesicles without rhEGF) were tested at the same minimal dilution of 1:10,000 as the minimal dilution used for evaluating rhEGF hydrogels. Furthermore, the minimum control of rhEGF in the colorimetric assay, in which cells are incubated only with culture medium, was used as a comparison to evaluate the differences in data. Then, when comparing the absorbance at 578 nm no statistically significant differences among the three different groups evaluated were observed (*n* = 10 per group, ANOVA *p*-value = 0.740), confirming no interference of Methocel^TM^ K4M in the colorimetric assay.

Figure 8b shows the results of the in vitro specific biological activity of all the tested samples, free and integrated rhEGF in CTAB-DELOS nanovesicles, both as liquid dispersion and gellified dosage form. As shown, for free rhEGF in dispersant media and its hydrogel formulation non-significant statistical differences were observed, then the biological activity of the protein was preserved when gellified. However, the specific bioactivity of the aqueous dispersion and hydrogel of rhEGF-DELOS nanovesicles increased as compared to the free rhEGF aqueous solution and its hydrogel formulation. These results are in agreement with previously reported rhEGF-DELOS nanovesicles where higher cell proliferation activity than free rhEGF were obtained at the same bulk concentration [8]. On the other hand, specific bioactivity of the rhEGF-DELOS nanovesicles-based hydrogel increased statistically significantly as compared to the aqueous solution of the same rhEGF-DELOS nanovesicles (Figure 8b). Because there is no change in specific bioactivity for free rhEGF in aqueous solution as compared to the hydrogel formulation, a plausible reason for the increase in specific bioactivity of rhEGF-DELOS nanovesicles gellified as compared to the aqueous solution could be that the stability of the binding of rhEGF to the nanovesicles surface in the presence of Methocel^TM^ K4M increased. Probably the nanoconjugate is more stable upon dilution in the presence of Methocel^TM^ K4M at large dilutions (>1:10,000 for 100 μg mL^−1^) used in the cell proliferation assay. Recently we do not have any experimental evidence for this finding and further study will be necessary to elucidate the reasons for the increased specific biological activity of rhEGF-DELOS nanovesicles in the Methocel^TM^ K4M hydrogel as compared to the aqueous dispersion.

## 4. Conclusions

This study evaluated the suitability and feasibility of novel DELOS nanovesicles as a semi-solid dosage form for topical administration. DELOS nanovesicles composed of two types of quaternary ammonium surfactants, CTAB and CTAC, which differ in the counterion used—bromide and chloride, respectively—were prepared by the eco-efficient one-step DELOS-SUSP methodology. Different dispersant media were screened in order to pursue more skin-tolerability of these DELOS nanovesicle systems, even in damaged skin. Accordingly, media such as water; acetate buffer, pH = 5.0; histidine buffer, pH = 7.0; and PBS buffer, pH = 7.4, were used for DELOS nanovesicles production, yielding dispersions with excellent physicochemical properties. Then, these liquid dispersions were gellified for the first time by adding 2% *w*/*w* Methocel^TM^ K4M obtaining DELOS nanovesicles-based hydrogels with good macroscopic appearance and appropriate rheological properties for the intended use. However, the presence of particles in the micrometric range was observed under optical microscopy in those semi-solid formulations that contained sodium acetate and PBS buffers, probably related to the presence of acetate and phosphate anions and its potential electrostatic interaction with the cationic surface charge of the nanovesicles. This change of particle size distribution was correlated with a significant viscosity increase of these formulations. Therefore, Methocel^TM^ K4M hydrogels containing DELOS nanovesicles formulated in water and histidine buffer were selected as the optimal ones. The integrity of these vesicles when being gellified has been proved by FRET fluorescence measurements and cryo-TEM imaging, maintaining their stability in the semi-solid form, which is a relevant factor not broadly considered in the literature. Additionally, these DELOS nanovesicles-based Methocel^TM^ K4M hydrogels have been demonstrated to be promising systems for the treatment of complex wounds. Indeed, the bioactivity of the recombinant human Epidermal Growth Factor (rhEGF) when formulated as rhEGF-DELOS nanovesicles-based hydrogels, has shown a considerable increase, probably due to the stabilization of the binding of rhEGF to the nanovesicles surface in the presence of Methocel^TM^ K4M.

## Figures and Tables

**Table 1:** Overview of the membrane components and dispersant media tested in the DELOS nanovesicles formulations under study.

Vesicle Membrane Components	Dispersant Media ^1^
Cholesterol:CTAB (CTAB-DELOS nanovesicles)	Water pH ca. 7.0 Sodium citrate buffer, pH = 5.0 (5 mM) Sodium acetate buffer, pH = 5.0 (5 mM) Histidine buffer, pH = 7.0 (5 mM) PBS buffer, pH = 7.4 (5 mM)
Cholesterol:CTAC (CTAC-DELOS nanovesicles)	Water pH ca. 7.0 Sodium citrate buffer, pH = 5.0 (5 mM) Sodium acetate buffer, pH = 5.0 (5 mM) Histidine buffer, pH = 7.0 (5 mM) PBS buffer, pH = 7.4 (5 mM)

^1^ All the samples contain 10% *v*/*v* of EtOH.

## Data Availability

Data can be requested by contacting the corresponding author.

## Supplementary Materials

The following supporting information can be downloaded at: https://www.mdpi.com/article/10.3390/pharmaceutics14010199/s1, Figure S1: Schematic representation of procedure for the preparation of DELOS nanovesicles by DELOS-SUSP technology; Figure S2: Particle size distribution of (a) CTAB-DELOS nanovesicles and (b) CTAC-DELOS nanovesicles in different dispersant media after 4 weeks post-production: water (blue ■); 5 mM acetate, pH = 5.0 (orange ▼); 5 mM histidine, pH = 7.0 (yellow ●); and 5 mM PBS, pH = 7.4 (green ▲). Figure S3: Macroscopic appearance of (a) CTAB-DELOS nanovesicles and (b) CTAC-DELOS nanovesicles in water (blue); 5 mM acetate buffer, pH = 5.0 (orange); 5 mM citrate buffer, pH = 5.0 (red); 5 mM histidine buffer, pH = 7.0 (yellow); and 5 mM PBS buffer, pH = 7.4 (green); Figure S4: Evolution of (a) CTAB-DELOS nanovesicles and (b) CTAC-DELOS nanovesicles over time on the zeta potential values with the effect of different dispersant media: water (blue); 5 mM acetate, pH = 5.0 (orange, horizontal line,―); 5 mM citrate, pH = 5.0 (red, waves, ≈); 5 mM histidine, pH = 7.0 (yellow, vertical line, |); and 5 mM PBS, pH = 7.4 (green, square, □); Figure S5: Cryo-TEM images of (a) CTAB-DELOS nanovesicles and (b) DiI/DiD-DELOS nanovesicles, prepared using CTAB as surfactant, in water; (c) macroscopic image of DiI/DiD-DELOS nanovesicles-based Methocel^TM^ K4M hydrogel; Figure S6: Colocalization analysis of DiI and DiD in DiI/DiD-DELOS nanovesicles-based Methocel^TM^ K4M hydrogel by confocal imaging, DiI/DiD-DELOS nanovesicles were prepared using CTAB as surfactant and water as media. (a) General view of the overlay images of excited DiI with 488 nm light (in green) and DiD with 633 nm light (in red). The yellow color represents the colocalization of the two dyes, indicating the presence of the two dyes in an unaltered vesicle; (b) magnification of the grey box revealing the abundance of particles smaller than 1 µm, indicating the homogeneous dispersion of DELOS nanovesicles in the hydrogel; Figure S7: Cryo-TEM images of rhEGF-DELOS nanovesicles prepared using CTAB as surfactant in 5 mM histidine (pH = 7) (a) non-gellified and (b) gellified with 2% *w*/*w* of Methocel^TM^ K4M. Gellified nanovesicles were previously diluted 1:100 in water for an adequate fixation and preservation of the hydrogel sample in the grid.
