# Supplementary material for: DELOS Nanovesicles-Based Hydrogels: An Advanced Formulation for Topical Use
Source: Pharmaceutics. 2022 Jan 15;14(1):199. doi: 10.3390/pharmaceutics14010199 (PMC8779640; doi:10.3390/pharmaceutics14010199)
Supplement: Supplementary file 1 [file pharmaceutics-14-00199-s001.zip › pharmaceutics-1504847-supplementary.pdf]

pharmaceutics

MDPI

Supplementary Materials: DELOS Nanovesicles-Based Hydrogels: An Advanced Formulation for Topical Use

Lídia Ballell-Hosa, Elisabet González-Mira, Hector Santana, Judit Morla-Folch, Marc Moreno-Masip, Yaima Martínez-Prieto, Albert Revuelta, Primiano Pio Di Mauro, Jaume Veciana, Santi Sala, Lidia Ferrer-Tasies and Nora Ventosa

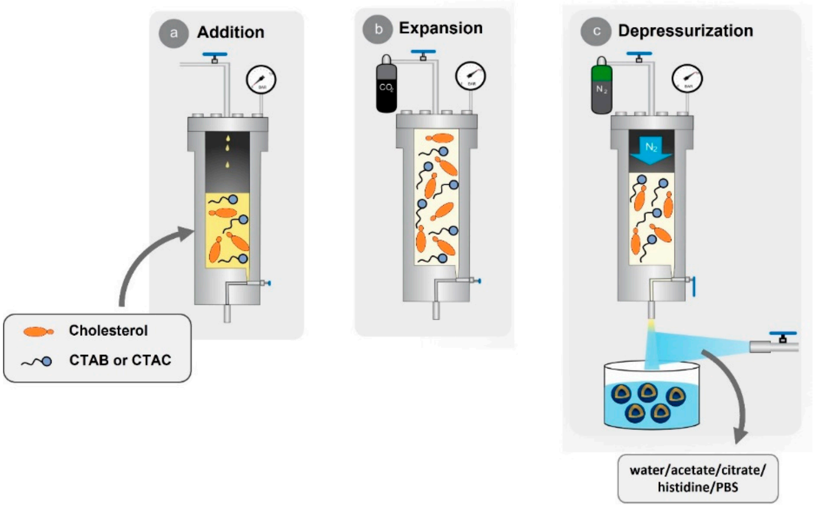

Figure S1. Schematic representation of procedure for the preparation of DELOS nanovesicles by DELOS-SUSP technology. The whole procedure includes (**a**) the loading of an ethanolic solution of the membrane compounds such as the cholesterol and the surfactant (CTAB or CTAC). For DiI/DiD-DELOS nanovesicle production, the fluorescent dyes (DiI and DiD) are also loaded together with the membrane compounds; (**b**) additon of compressed CO_2 and formation of CO_2 expanded solution, and (**c**) despressurization into an aqueous solution. In this work, the aqueous solution can be water; 5 mM sodium acetate buffer, pH = 5.0; 5 mM sodium citrate buffer, pH = 5.0; 5 mM histidine buffer, pH = 7.0; and 5 mM PBS buffer, pH = 7.4. Additionally, to produce the rhEGF-DELOS nanovesicles, in this final step rhEGF is dissolved in 5 mM histidine buffer pH = 7.0.

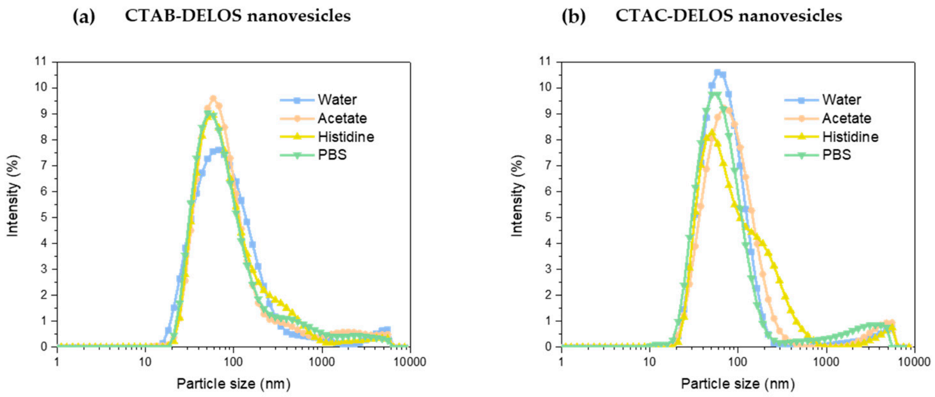

Figure S2. Particle size distribution of (**a**) CTAB-DELOS nanovesicles and (**b**) CTAC-DELOS nanovesicles in different dispersant media after 4 weeks post-production: water (blue ■); 5 mM acetate, pH = 5.0 (orange ▽); 5 mM histidine, pH = 7.0 (yellow ●); and 5 mM PBS, pH = 7.4 (green △).

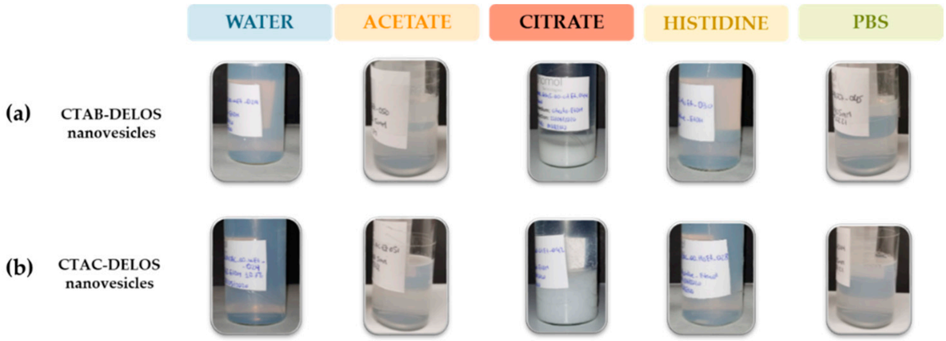

Figure S3. Macroscopic appearance of (**a**) CTAB-DELOS nanovesicles and (**b**) CTAC-DELOS nanovesicles in water (blue); 5 mM acetate buffer, pH = 5.0 (orange); 5 mM citrate buffer, pH = 5.0 (red); 5 mM histidine buffer, pH = 7.0 (yellow); and 5 mM PBS buffer, pH = 7.4 (green).

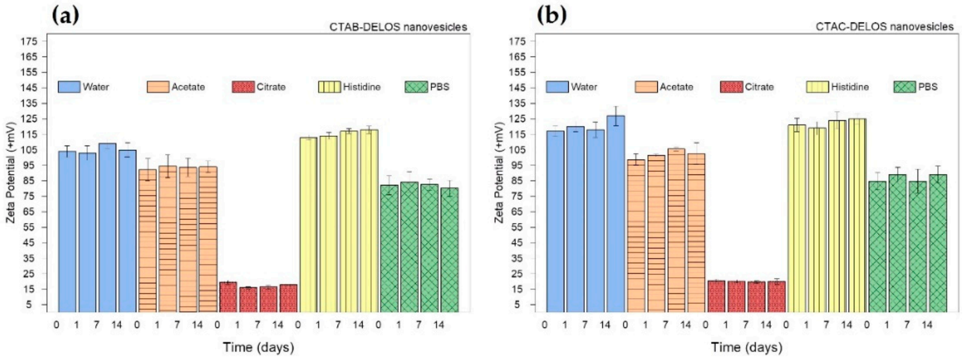

Figure S4. Evolution of (**a**) CTAB-DELOS nanovesicles and (**b**) CTAC-DELOS nanovesicles over time on the zeta potential values with the effect of different dispersant media: water (blue); 5 mM acetate, pH = 5.0 (orange, horizontal line, —); 5 mM citrate, pH = 5.0 (red, waves, ≈); 5 mM histidine, pH = 7.0 (yellow, vertical line, |); and 5 mM PBS, pH = 7.4 (green, square, □).

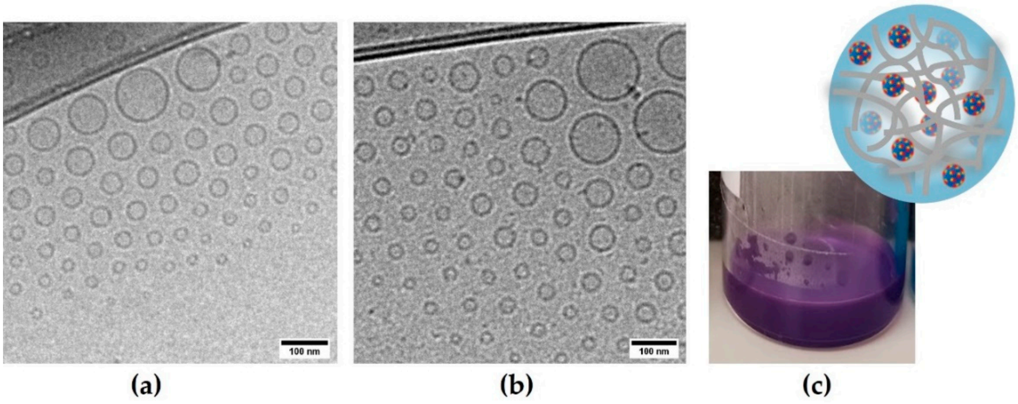

Figure S5. Cryo-TEM images of (**a**) CTAB-DELOS nanovesicles and (**b**) DiI/DiD-DELOS nanovesicles, prepared using CTAB as surfactant, in water; (**c**) macroscopic image of DiI/DiD-DELOS nanovesicles-based Methocel™ K4M hydrogel.

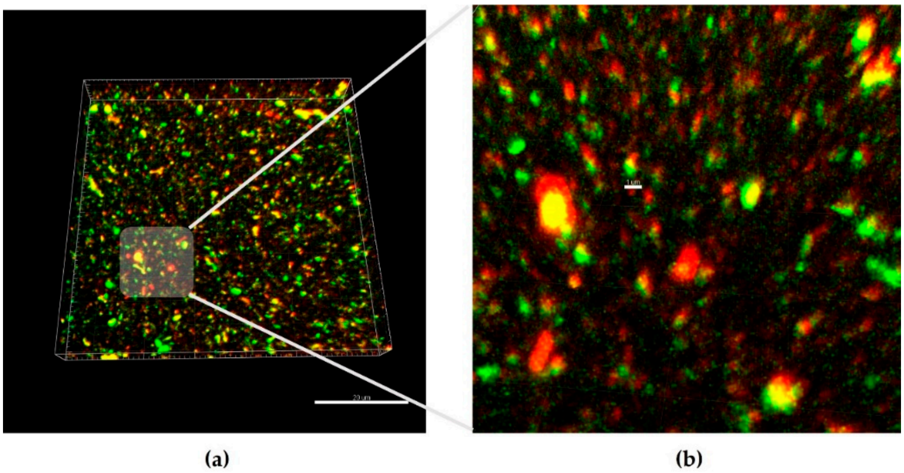

Figure S6. Colocalization analysis of DiI and DiD in DiI/DiD-DELOS nanovesicles-based Methocel™ K4M hydrogel by confocal imaging, DiI/DiD-DELOS nanovesicles were prepared using CTAB as surfactant and water as media. (**a**) General view of the overlay images of excited DiI with 488 nm light (in green) and DiD with 633 nm light (in red). The yellow color represents the colocalization of the two dyes, indicating the presence of the two dyes in an unaltered vesicle; (**b**) magnification of the grey box revealing the abundance of particles smaller than 1 micrometer, indicating the homogeneous dispersion of DELOS nanovesicles in the hydrogel.

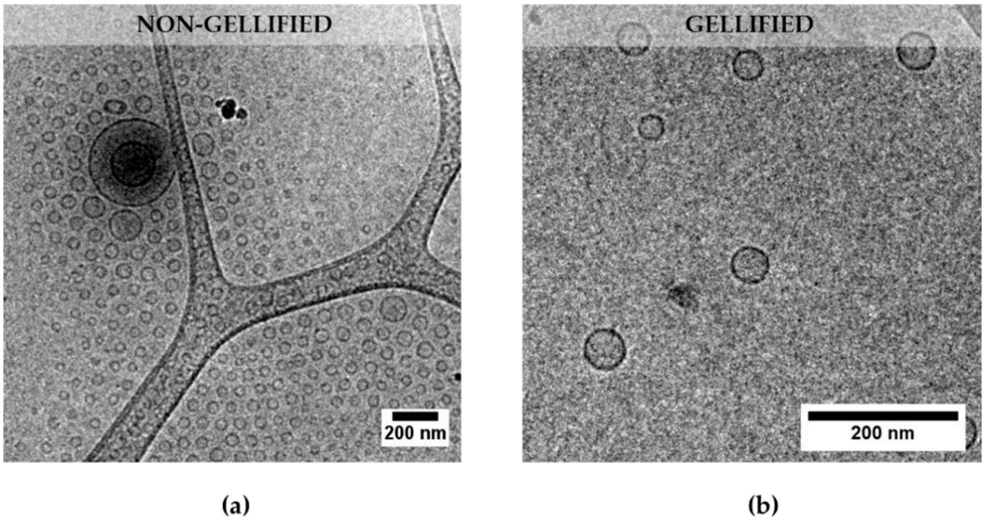

Figure S7. Cryo-TEM images of rhEGF-DELOS nanovesicles prepared using CTAB as surfactant in 5 mM histidine (pH = 7) (**a**) non-gellified and (**b**) gellified with 2% w/w of Methocel™ K4M. Gellified nanovesicles were previously diluted 1:100 in water for an adequate fixation and preservation of the hydrogel sample in the grid.
